# Physical activity equivalent labeling vs. calorie labeling: a systematic review and meta-analysis

**DOI:** 10.1186/s12966-018-0720-2

**Published:** 2018-09-14

**Authors:** Shirin Seyedhamzeh, Minoo Bagheri, Abbas Ali Keshtkar, Mostafa Qorbani, Anthony J. Viera

**Affiliations:** 1 Food and Nutrition Policy, Department of Community Nutrition, School of Nutritional sciences and Dietetics, Tehran University of Medical Sciences, Tehran, Iran; 2 Students’ Scientific Research Center, Tehran University of Medical Sciences, Tehran, Iran; 3 PhD student in Nutrition, Department of Community Nutrition, School of Nutritional Sciences and Dietetics, Tehran University of Medical Sciences, Tehran, Iran; 4 MD, MPH, PhD of Epidemiology, Department of Health Sciences Education Development, School of Public Health, Tehran University of Medical Sciences, Tehran, Iran; 5 PhD of Epidemiology, Non-communicable Diseases Research Center, Alborz University of Medical Sciences, Karaj, Iran; 6 MD, MPH, Professor and Chair, Department of Community and Family Medicine, Duke University, Durham, NC USA; 7 No 44, Hojjat-dost Alley, Naderi Street, Keshavarz Boulevard, Tehran, 1416-643931 Iran

**Keywords:** Physical activity equivalent labeling, Food labeling, Calorie labeling, Meta-analysis

## Abstract

**Background:**

Many countries are trying to identify strategies to control obesity. Nutrition labeling is a policy that could lead to healthy food choices by providing information to consumers. Calorie labeling, for example, could lead to consumers choosing lower calorie foods. However, its effectiveness has been limited. Recently, physical activity equivalent labeling (i.e., displaying calories in terms of estimated amount of physical activity to burn calories) has been proposed as an alternative to the calorie-only label. The aim of this review was to identify and evaluate the published literature comparing effects on health behavior between physical activity equivalent labeling and calorie-only labeling.

**Method:**

We searched the following databases: Pubmed/medline, Scopus, Web of science, Agris, Cochrane library, Google Scholar. We also searched along with reference lists of included articles. Articles that were published between 1 January 2000 and 31 October 2016 were eligible for inclusion provided they reported on studies that examined the effects of both types of labeling and included at least one outcome of interest. Mean and standard deviations of the included results were combined using a fixed-effect model. The difference in calories purchased between people exposed to physical activity labeling and calorie-only labeling was calculated as weighted mean difference by using a fixed-effect model.

**Result:**

The difference of calories ordered between physical activity label and calorie label groups was not statistically significant (SMD: -0.03; 95% CI: -0.13, 0.07). The difference of calories ordered between physical activity label and calorie label according to real vs unreal (e.g. web-based) condition was 65 Kcal fewer in real-world settings.

**Conclusion:**

Physical activity calorie equivalent labeling in minutes does not significantly reduce calories ordered compared to calorie-only labeling.

**Electronic supplementary material:**

The online version of this article (10.1186/s12966-018-0720-2) contains supplementary material, which is available to authorized users.

## Background

Obesity is recognized as a significant threat to health. The increasing incidence of obesity, beyond imposing substantial costs, has been a source of concern for policy makers in health programs. Key factors in the development of obesity are unhealthy diets and low levels of physical activity. Raising awareness of the influence of diet and physical activity on weight can be an effective strategy to combat obesity [1, 2].

Recent studies have suggested that caloric restriction by 30% could reduce the incidence of type 2 diabetes, cardiovascular disease and cancer [3–5]. The Comprehensive Assessment of the Long-term Effects of Reducing Intake of Energy (CALERIE) research program investigated the effects of 25% energy restriction in people without obesity for 2 years. The results of this study showed reductions in body weight by 11.5%, in fat free mass by 4.3%, and in fat mass by 23% at month 12 [6, 7]. Calorie restriction and exercise not only contributed to weight loss but they also had a significant effect in the reduction of oxidative damage to DNA and RNA [7, 8].

Many developed countries are trying to identify strategies to reduce the burden of overweight and obesity [8, 9]. One such strategy is providing labeling and consumer information [10], although studies at the population level have shown inconsistent results on the effects of nutrition labeling [11]. Calorie labeling on menus might help combat obesity and overweight by influencing consumers’ food purchasing and eating behaviors. Although calorie labeling has been found to have limited effectiveness on changing behaviors [12], consumers appear to want calorie menu labeling [13–15]. Physical activity calorie equivalent labeling is a type of nutrition labeling that might have more influential effects on food choice than other label formats [16]. By using a label that provides the amount of physical activity represented by the calories in a food item, people could more easily balance their diet with their physical activity level [17]. Symbols are more understandable than numerical information and people’s behavior might be affected by them [18], so investigators are trying to find whether there is any relationship between this new kind of label and food choices. Evidence appears mixed as to whether this kind of labeling leads to changes in consumer’s behaviors [19–25].

Since no published meta-analysis has assessed the effectiveness of physical activity equivalent labeling and calorie labeling and previous individual studies in real and unreal settings showed controversial results, we systematically reviewed the published literature comparing the difference of effects on health behavior between physical activity equivalent labeling and calorie-only labeling. To our knowledge there is no meta-analysis on this topic.

## Methods

### Study selection

Based on our primary search there were few studies which compared calorie labeling and physical activity equivalent labeling.

#### Inclusion criteria

We included studies that compared calorie labeling and physical activity labeling, with either population or non-population based data without any restriction on race, education and socioeconomic status, types of food. We included studies in various settings including restaurants and schools. Other criteria used for study selection were study population aged > 18 years and inclusion of an intervention of physical activity label using either mile or in minutes.

#### Exclusion criteria

The studies were excluded if they were qualitative, commentaries, letters, or conference proceedings.

#### Interventions and outcomes of interest

Outcomes of interest were calories purchased. The PICOS (population, intervention, comparator, outcome, setting) criteria used to perform the systematic review are outlined in Additional file 1: Table S1.

Calorie label as interested interventions was defined as labels that show the amount of calorie in foods and minutes or miles need to burn the calories of food ordered, respectively.

The effect of calorie labeling and physical activity labeling was examined on calories ordered. Calorie ordering as outcome of interest was defined as the amount of calorie of the foods that customers order after considering menu in the restaurants.

### Quality assessment

We considered assessing the quality of the included studies by using the Cochrane assessment tool [26]. However, considering that many of the studies were conducted using hypothetical scenarios or non-real-world settings existing quality tools might not yield a proper assessment of the studies. Therefore, a quality assessment tool was designed one of the authors (AAK). Quality was scored based on setting (real, unreal design), randomization, mentioning inclusion and exclusion criteria, generalization, quality of participants’ responses, implementing pilot phase, enough variety of menu, assessing physical activity after intervention, and assessing factors and their effects on the primary outcome. Then, first and second authors classified studies into three categories: high risk of bias, low risk of bias and unclear (Appendix 1).

### Search strategy

We searched the following databases: Pubmed/medline, Scopus, Web of science, Agris, Cochrane library, Google Scholar. We also searched reference lists of included articles. Articles that were published between 1 January 2000 and 31 October 2016 were included. We searched Google Scholar and Agris to find grey literature. Key words were obtained from Mesh, Emtree or extracted from related articles. Keywords which were obtained from Emtree or Mesh were included in our search strategy without any changes. Since our aim was to compare the effect of two different types of labeling (calorie labeling and physical activity equivalent labeling) on food choice, the study syntax was formed from two components. The first component referred to label (calorie and physical activity) including: “food label”, “nutrition label”, “menu label”, “food marking”, “food packing”, “food wrapping”, “calorie converter”, “eat label”, “nutrient label”, “nutrient content”, “food away from home”, “traffic light”, “PACE”, “motor activity” “physical activity”, “locomotor activity”, “exercise”, “energy expenditure”, “walk” and the second component included “caloric restriction”, “diet”, “low-calorie”, “low calorie diet”, “energy intake”, “caloric intake restriction”, “calorie”, “kilocalorie”, “food energy”, “K-Cal”, “Kcal”, “meal low calorie”, “meal low-calorie”, “food order”, “food consume”, “food consumption”, “food decision”, “diet selection”, “diet decision”, “food desire”, “diet desire”, “food choose”, “diet choose”, “appetite regulation”, “feed intake”, “feeding methods”, “food ingestion”, “food intake regulation”, “food intake”, “food uptake”, “food Preference”, “selection Food”, “food choice”, “behavior eating”, “nutrition behavior”. Our search strategy is shown in Appendix 2.

Studies were selected without language restriction, and non-English language publications were translated into English. First and second authors screened the title and abstract independently and the screened results were assessed. If there were any disagreements in data extraction, a decision was made by consensus. Full texts of included articles were identified. One author (SS) extracted data. The information extracted from the full-text of the included articles included: country, type of setting (school, restaurant or web based design), study design (experimental or quasi-experimental), participant characteristics (age, sex, BMI) and quantitative data needed for meta-analysis (samples size, means and standard deviations of calorie label groups and physical activity label groups).

The averages of differences in calories ordered (mean ± SD(between the two groups (calorie label and physical activity label) were extracted. Then the value of standardized mean difference (SMD) using the means and standard deviations (SD) of two groups (calorie label and physical activity label) were calculated as follow:

$$ {\mathsf{Cohen}}^{'}\mathsf{s}\ \mathsf{d}={\mathsf{Mean}}_{\mathsf{1}}-{\mathsf{Mean}}_{\mathsf{2}}/\mathsf{SD}\ \mathsf{pooled} $$

$$ \mathsf{SD}\ \mathsf{pooled}=\surd \left[\left({\left({\mathsf{s}}_{\mathsf{1}}\right)}^{\mathsf{2}}+{\left({\mathsf{s}}_{\mathsf{2}}\right)}^{\mathsf{2}}\right)/\mathsf{2}\right] $$

Authors of studies with unclear information were contacted. If we did not receive a response after contacting the corresponding author for three times over 6 weeks, we excluded the study.

We followed the PRISMA checklist for this study [27] (Additional file 2).

### Statistical analysis

Data were analyzed using STATA 12 (StataCorp, College Station, TX, USA). To compare the effect of interventions we used Cohen’s d approach [28]. In this approach, Cohen’s d which is also known the standardized mean difference (SMD) was calculated using the following formula:

$$ \mathsf{SMD}={\mathsf{Mean}}_{\mathsf{1}}-{\mathsf{Mean}}_{\mathsf{2}}/\mathsf{Standard}\ \mathsf{deviation}\ \mathsf{pooled} $$

$$ \mathsf{Standard}\ \mathsf{deviation}\ \mathsf{pooled}=\surd \left[\left({\left({\mathsf{S}}_{\mathsf{1}}\right)}^{\mathsf{2}}+{\left({\mathsf{S}}_{\mathsf{2}}\right)}^{\mathsf{2}}\right)/\mathsf{2}\right] $$

In this formula Standard deviation pooled (SD) is a weighted average of standard deviations for two groups. The individual standard deviations are averaged, with more “weight” given to larger sample sizes. Meta-analysis was performed to estimate the pooled SMD of the effect of physical activity equivalent labeling vs. calorie labeling on calories ordered. The Q Cochrane test and I^2^ statistics were used to assess heterogeneity between studies. The I^2^ statistics less than 25%, 25–50% and more than 50% were considered as little, moderate and severe heterogeneity, respectively. A fixed effects met-analysis method was used to pool the estimated SMD. The forest plot was used to schematically present the pooled SMD and its 95% CI. To assess the source of heterogeneity, subgroup analyses (according to quality score, BMI, age, sex and study setting) were manipulated. Publication bias was assessed by Egger’s regression asymmetry test [29] and trims and fills method [30]. Also we used Egger’s test to assess the existence of unpublished data. The studies that reported physical activity labeling in minutes and miles were separately analyzed. We also conducted another sensitivity analysis to assess the influence of the label types and the study quality. Statistical significance was considered at *p* < 0.05. This study was registered at http://www.crd.york.ac.uk/PROSPERO/ as CRD42017051697.

## Results

**Fig. 1:**
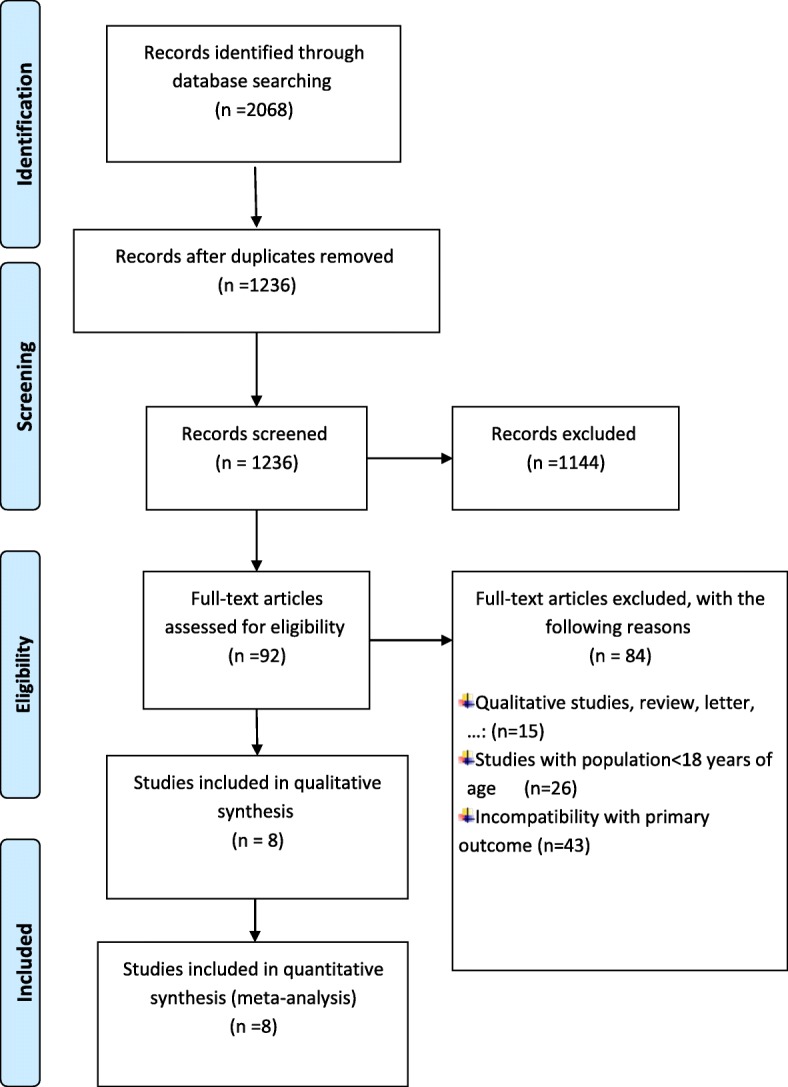
Flow chart of the selection study

A total of 2068 article titles were identified by database searching. After removing duplicates, 1236 articles were screened based on the study inclusion criteria. Of 92 full-text article evaluated, 84 were excluded with the following reasons: qualitative studies, review, letter and commentary (*n* = 15), studies with population aged less than 18 years (*n* = 26) and incompatibility with primary outcome (*n* = 43).Finally, 8 articles were eligible for inclusion. One of them conveyed physical activity in miles format [22]. Two studies used physical activity labels in mile and minute (min) formats [19, 20], and the rest displayed the physical activity label in minutes [21, 23–25, 31]. Figure 1 presents the flow chart of the study selection.

The settings of the studies varied. Two studies [21, 23] were conducted in real world settings, and the rest [19, 20, 22, 24, 25, 31] were conducted in web based and hypothesis format (Table 1).

**Table 1:** Characteristics of the included studies

First author (year)	Country	Mean age	Mean BMI	% Female	Setting real-unreal	Sample size	Mean ± SD^a^ physical activity label in Mile	Mean ± SD physical activity label in Minute	Mean ± SD calorie label
Lee MS (2016) [22]	USA	20.44	24.15	78.5	Unreal	428	1045.51 ± 626.09	NA	1022.29 ± 547.76
James A (2015) [21]	USA	21.95	24.15	56.25	Real	201	NA	763 ± 311.74	827 ± 309.66
Antonelli R (2015) [19]	USA	38.67	28	71.33	Unreal	634	1371 ± 828	1334 ± 756	1329 ± 755
Reale S (2016) [24]	UK	50.52	41.17	62.29	Unreal	86	NA	161.07 ± 65.27	601.03 ± 254.23
Pang J (2013) [31]	Canada	20.55	NA	66	Unreal	106	NA	309.8 ± 59	301.6 ± 58.9
Dowray S (2013) [20]	USA	44	28.43	86	Unreal	602	826.29 ± 539.18	916.15 ± 664.45	927.05 ± 681.74
Shah M (2016) [25]	USA	33.9	29.6	61.25	Unreal	245	NA	768.76 ± 385.46	773.33 ± 382.57
Platkin C (2014) [23]	USA	21.9	28.7	100	Real	40	NA	1000.5 ± 439.16	1077 ± 509.82

^a^*SD* Standard Deviation

**Fig. 2:**
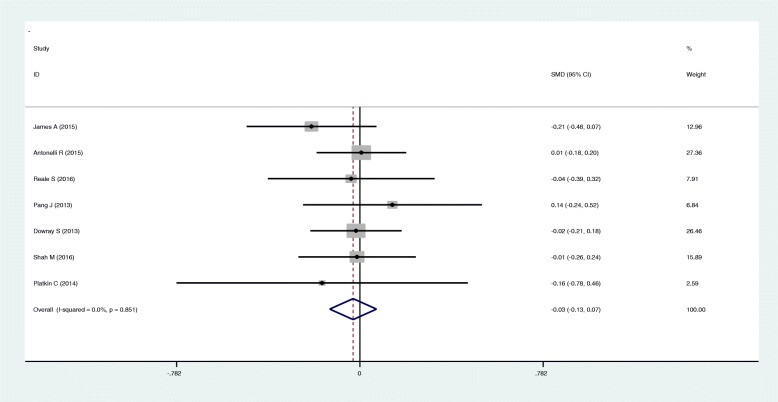
Forest plot of mean difference in physical activity label (min) versus mean in calorie label. The pooled SMDs were calculated by using a fixed-effect model

**Fig. 3:**
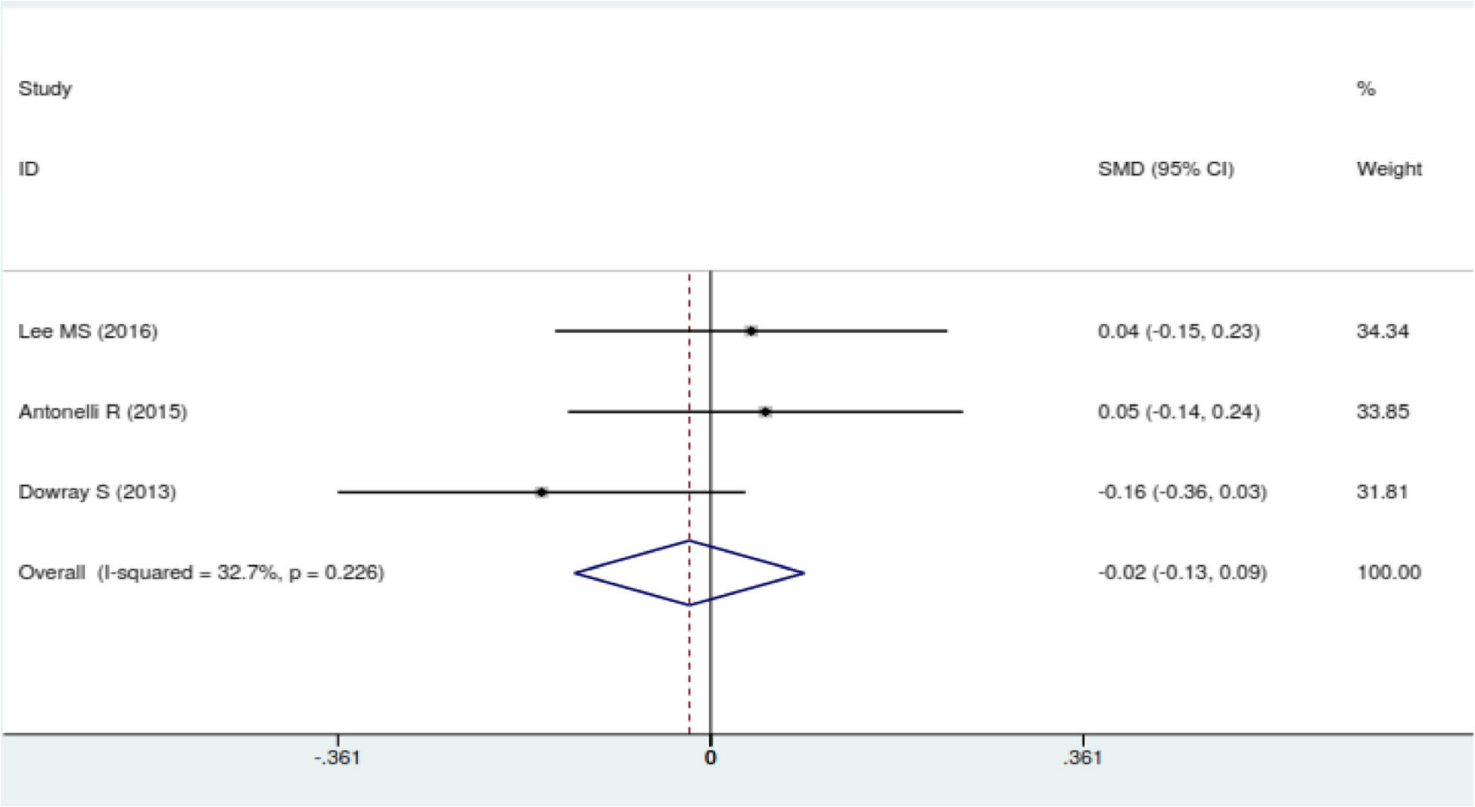
Forest plot of mean difference in physical activity label (mile) versus mean in calorie label. The pooled SMDs were calculated by using fixed-effect

In meta-analysis, there was a slight but non-significant reduction in calorie ordering at the point of food selection in groups shown physical activity labeling in minutes vs calories only(SMD: -0.03; 95%CI: -0.13, 0.07) (Fig. 2). There was no difference in calories ordered among people shown physical activity labeling in miles vs calories only (SMD: -0.02; 95%CI: -0.13, 0.09) (Fig. 3).

**Fig. 4:**
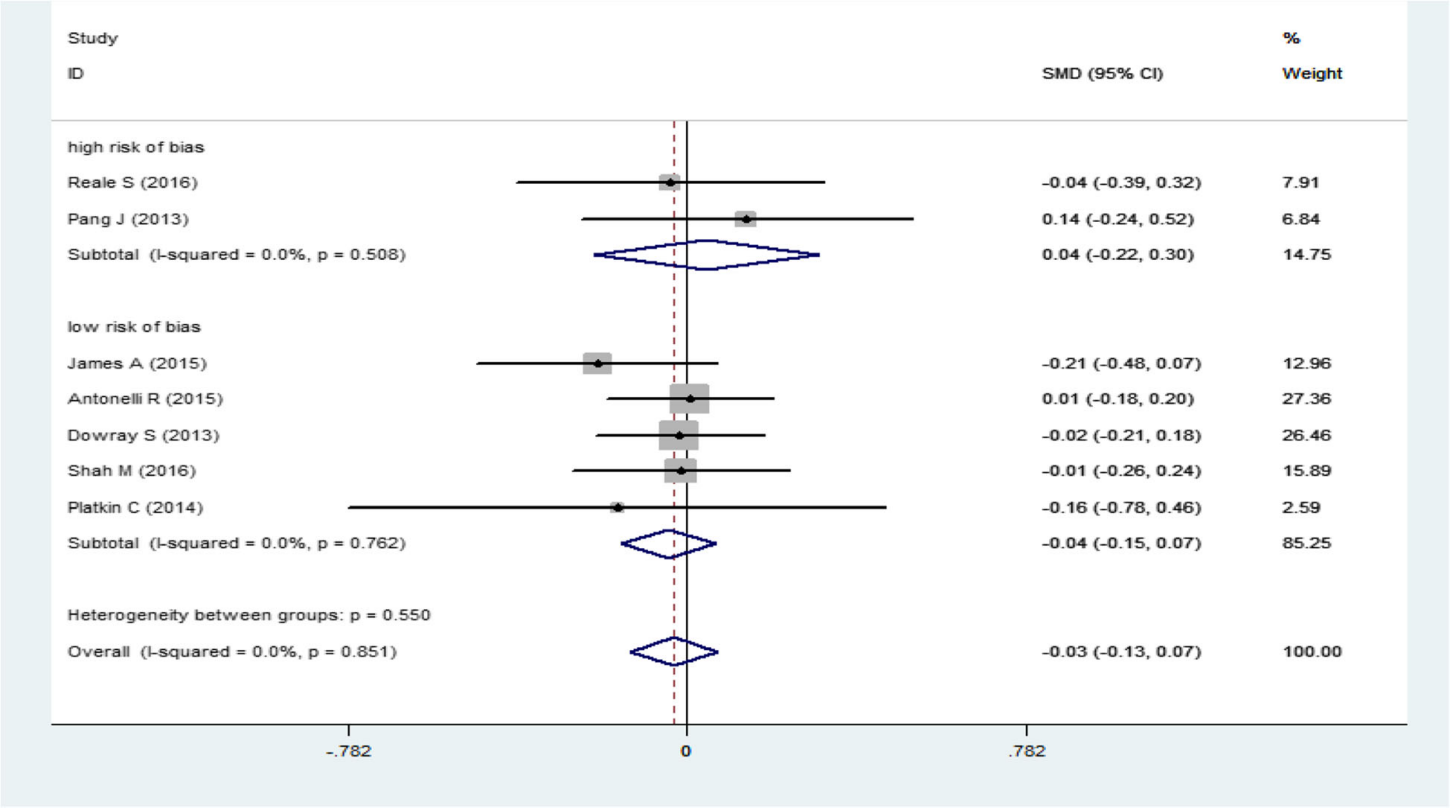
Forest plot for the association of energy order with quality assessment

Subgroup analysis of calories ordered in physical activity label and calorie label based on quality assessment is shown in Fig. 4. In studies with high risk of bias the calories ordered were slightly increased (SMD: 0.04; 95%CI: -0.22, 0.30). However, in low risk of bias studies the amount of calories ordered was slightly decreased (SMD: -0.04; 95%CI: -0.15, 0.07).

**Fig. 5:**
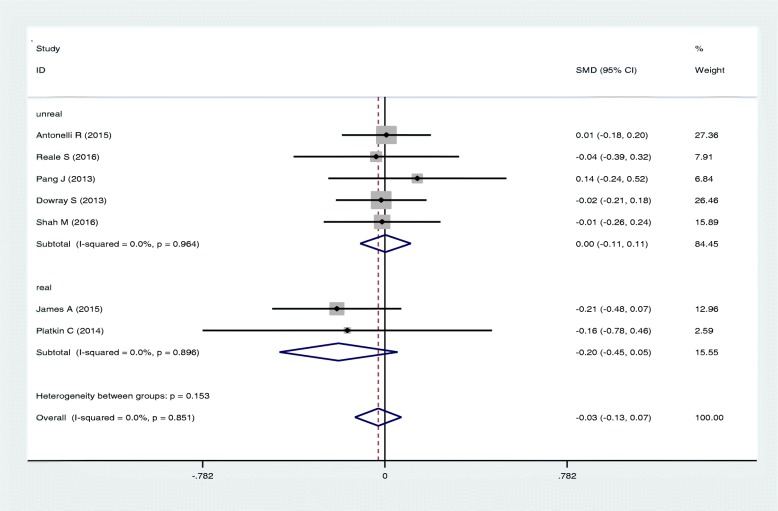
Forest plot for the association of energy order with study setting (real and unreal)

Calories ordered was not associated with either unreal setting (SMD: - 0.003; 95%CI:-0.106, 0.111), or real-world setting (SMD: -0.198; 95%CI: -0.128, 0.071) (Fig. 5).

Subgroup analysis of calories ordered by quality, BMI, age, percentage of female, and setting of studies is shown in Table 2.

**Table 2:** Subgroup analysis on mean of energy order by quality, BMI, age, percentage of female, and setting of studies

Subgroup	SMD^a^	[95% Conf. interval]	I^2^	Q^b^	*P*-value
Quality					
High	−0.041	− 0.241, − 0.038	0.0%	1.86	0.762
low	0.045	−0.215, 0.305	0.0%	0.44	0.508
BMI					
< 28.5	−0.044	− 0.166, 0.078	0.0%	1.66	0.436
≥ 28.5	−0.034	−0.228, 0.160	0.0%	0.19	0.732
Age					
< 28	−0.095	−0.306, 0.116	4.9%	2.10	0.349
≥ 28	−0.009	−0.123, 0.104	0.0%	0.06	0.997
%Female					
< 68.5	−0.050	−0.202, 0.101	0.0%	2.25	0.522
≥ 68.5	−0.012	−0.145, 0.121	0.0%	0.26	0.879
Setting					
Real	−0.198	−0.452, 0.055	0.0%	0.02	0.896
Unreal	0.003	−0.106, 0.111	0.0%	0.59	0.964
Total	−0.029	−0.128, 0.071	0.0%	2.65	0.851

^a^Standardized Mean Difference

^b^Heterogeneity Statistics

**Fig. 6:**
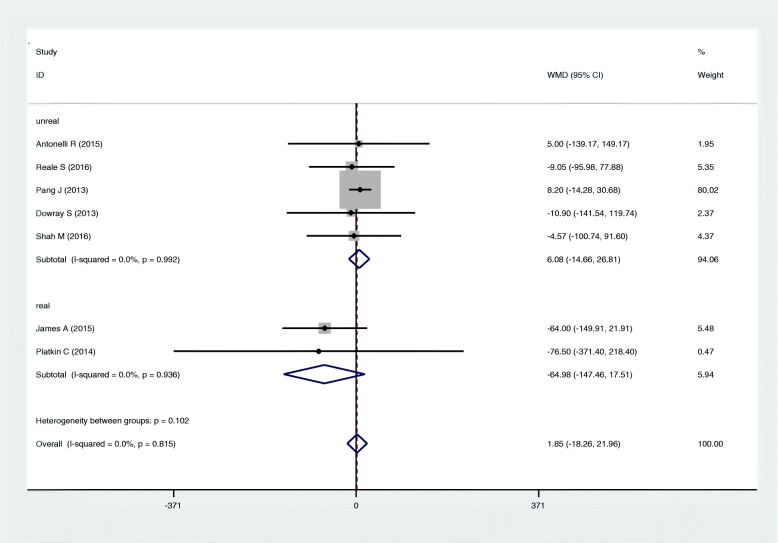
Forest plot for the amount of energy reduction with study setting (real world and unreal world)

The effect of physical activity label on calorie reduction is shown in Fig. 6. In real condition studies, the average reduction in calories ordered was 65 cal among people viewing physical activity labels.

Publication bias and sensitivity analysis were evaluated based on quality assessment. Findings from the sensitivity analysis in low risk of bias studies revealed that excluding any single study did not alter the main findings (Table 3).

**Table 3:** Sensitivity analysis in high quality studies

Selected study	SMD^a^	95%CI	Z	*P*-Value	I^2^
1	−0.012	− 0.129 0.106	0.20	0.844	0.0%
2	−0.064	−0.195 0.067	0.96	0.339	0.0%
3	−0.053	−0.183 0.078	0.79	0.428	0.0%
4	−0.048	−0.168 0.072	0.79	0.432	0.0%
5	−0.038	−0.147 0.072	0.67	0.502	0.0%

^a^Standardized Mean Difference

**Fig. 7:**
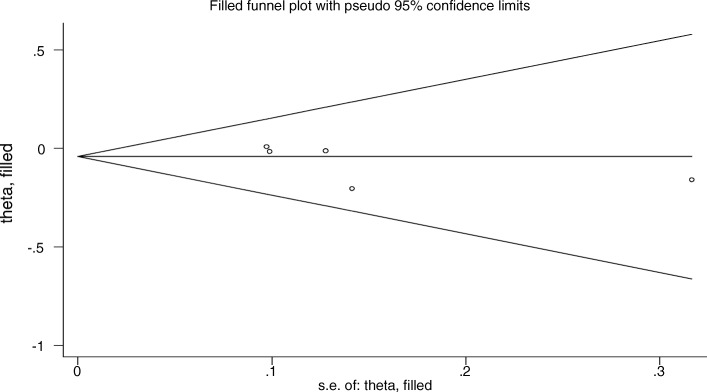
publication bias assessment conducted by trim and fill method in high quality studies

On the same basis, no evidence of publication bias was observed (*P* = 0.304 in Egger’s test, *P* = 0.762 in trim and fill method). The plot of trim and fill method is shown in Fig. 7. Moreover, Egger’s test showed that there was not any unpublished data.

## Discussion

The main finding of this systematic review is that using physical activity labeling (e.g., minutes of walking to burn calories) contributes to a slight but non-significant reduction in amount of calories in food ordering. Subgroup analysis showed no differences in these results based on age, sex, or BMI. There also was no difference based on setting:, the “real-world” studies appeared to reduce calories ordered by 65 cal, but this result was not statistically significant. To the best of our knowledge, this study is the first meta-analysis that compares the effects of calorie-only labeling with physical activity labeling.

It should be taken into account that studies in real world settings provided more precise and powerful findings than those in unreal settings. In one real setting study, the calories ordered in the physical activity label group were lower than that of in the calorie label group, yet the observed association failed to reach statistical insignificance. This finding, in the study by Platkin et al. [23], was mainly observed in overweight and obese females, although the sample size was low.

According to the results of the research by Shah et al. [25], there is no significant difference in calories ordered between people exposed to the physical activity label (minutes) vs the calorie label groups in Hispanic population, although the participants ordered fewer calories in the physical activity label group. The result of this study is consistent with those of the studies by Viera et al. [19] and Dowray et al. [20]. However, in the study by Pang and Hammond [31], the amount of calories ordered in the group of physical activity label was non-significantly greater than that of in the calorie label group. The inconsistency in results was attributed to the differences in food choices and the format of the label used in studies. Particularly, in the latter study, snacks (muffins and donuts) were used with a relatively small variation and the amount of physical activity in each case was written in minutes.

The number of studies conveying physical activity in miles was low, but in two studies [19, 25] the average of calories ordered among people shown miles was higher than that of those in the calorie label group. In the hypothetical scenario study conducted by Dowray et al. [20], people shown physical activity labels in miles ordered fewer calories than those shown physical activity in minutes, although the difference was not statistically significant.

A combination of many factors including education level, ethnicity, and socio-economic status influence people’s food choices [32]. Several studies assessed in this systematic review suggested that education in nutrition could result in healthier food choices [19, 20, 22, 24, 31].

In one study, both calorie and physical activity labels had less effect on exercise-related outcomes [22]. However, findings from other studies showed that the physical activity label is more likely to contribute to lower-calorie food choices and increased physical activity levels [16, 19]. Among low-income African American adolescents, physical activity but not calorie information had a significant effect in reducing the purchase of sugar-sweetened beverages [33]. On the other hand, among Hispanics, and in participants from Western and Mid-Western geographic regions with household income of less than $35,000, no significant associations were reported among consumers for these outcomes [19].

Another question is whether the label’s appearance as a schematic representation can make a difference in people’s preferences. In a study that used eye-tracking, a schematic image that depicted minutes to run on the nutrition factsheet was felt to be trustworthy [34].

Although food labeling is not a new strategy for educating consumers, researchers continue looking for the most effective methods. This issue is becoming increasingly important as we face the obesity outbreak and increased diversity of food products. In addition, since various factors such as taste, price, culture, and knowledge play roles in food choices making the ability to influence food decisions even more difficult. Physical activity labeling of food products has been criticized in several ways. One of the criticisms of the use of physical activity label is the failure in incorporating the base metabolic rate [35], which can cause a misleading effect on how our bodies use energy. The average person may think that all calories must be burned by “extra” physical activity making it appear nearly impossible to burn so many calories [37]. On the other hand Cramer et al. [17] suggests that if people get aware of the amount of activity they need to burn the energy, they can be encouraged to be more physically active. It should be noted that this meta-analytic review has been conducted on studies performed only on adults. The hypothetical scenario study by Viera and Antonelli [38] in which the participants selected foods items for their children was not included in this review. We found only one eligible study that examined potential outcomes among children, but in this study the parents were asked about food ordering for their children [38]. Therefore, we included the results for the parents [19]. Similar studies should be conducted on younger ages in order to assess their preferences. The results of these studies may be used to encourage families toward healthier food choices. Part of the above encouragement may be done through education [36].

This study has several strengths. First, we included studies that had large sample sizes. Secondly, most studies included in our meta-analysis were adjusted for confounding factors. Third, we performed subgroup analysis by BMI, percentage of females, age and setting of studies.

Our review also has several limitations. First, the number of included studies was small. In particular, more real-world studies are needed to have a better understanding of labeling effects. Secondly, in this study, the effect of two types of labeling on calorie purchasing was explored. However, the amount of consumption was not addressed. This was mainly due to the limited real-world studies included in this review. Third, because of the low number of studies, we could not compare the calorie order between the physical activity label groups in miles and minutes. More studies are needed to assess the effectiveness of these two types of labeling. Fourth, search for unpublished studies was not conducted.

## Conclusion

In summary, physical activity calorie equivalent labeling compared to calorie-only labeling did not significantly reduce calories ordered. In this study we included 8 studies all of which were conducted in high-income countries and in which races including black, white, and mixed were investigated. Therefore, we expect that our results could be generalized to different populations from high-income countries. Further original research in middle- and low- income countries is necessary to confirm whether these findings are similar in such settings. Also, more research is needed to examine whether physical activity labeling influences exercise behaviors.

### Additional files

Additional file 1: **Table S1.** PICOS (population, intervention, comparator, outcome, setting). (DOC 29 kb)

Additional file 2: PRISMA 2009 Checklist. (DOC 63 kb)

## Figures and Tables

**Fig. 8:**
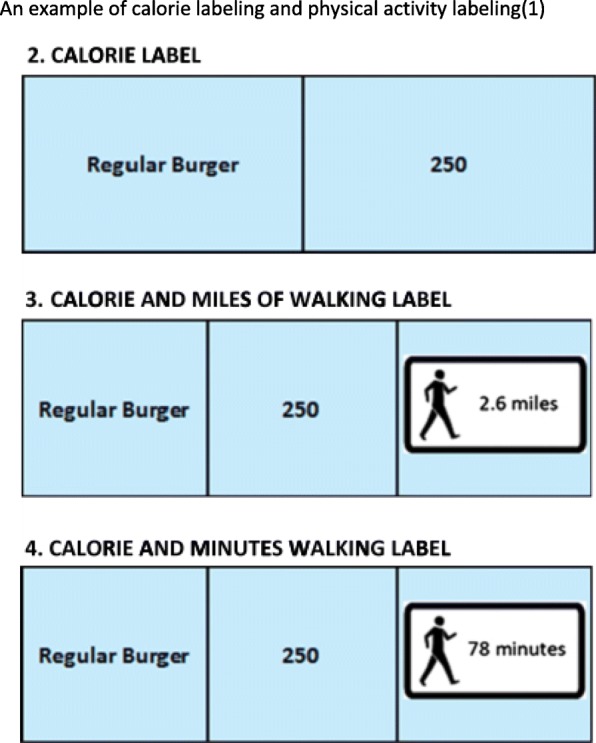
An example of calorie labeling and physical activity labeling [20]

## Appendix 1

**Table 4:** Quality assessment tool

Num	Question item	Criteria	Answer
1	Is the research has been conducted in real world?		Low Risk of Bias High Risk of Bias Unclear
2	Is the randomization method described?	Age, Education, Socio-economic status, BMI	Low Risk of Bias High Risk of Bias Unclear
3	Are inclusion criteria have been mentioned?	Age, BMI, …	Low Risk of Bias High Risk of Bias Unclear
4	Are exclusion criteria have been mentioned?	Age, BMI, physical activity, dieting, Special diets such as vegetarian, pregnancy,… At least, 2 exclusion criteria should be mentioned.	Low Risk of Bias High Risk of Bias Unclear
5	Is the study generalizable?	According to Race, BMI, Age Students, Academic people	Low Risk of Bias High Risk of Bias Unclear
6	Are there any criteria to assess quality of participants’ responses?	At least one of these criteria shows quality assessment of responses: - Very quick answers - Uncompleted data related to outcome - Assessment of hunger, desire to eat, before food selection and the amount of total calorie intake (in experimental researches) - Including incentive for completeness	Low Risk of Bias High Risk of Bias Unclear
7	Is the questionnaire implemented in pilot phase?	Consumer views about menu diversity	Low Risk of Bias High Risk of Bias Unclear
8	Does the menu have enough variety?	According to carbohydrate, protein, and beverages (at least 1 sweetened beverages) - If one of the groups not included in the menu, it should be mentioned as high risk of bias.	Low Risk of Bias High Risk of Bias Unclear
9	Are the differences of factors and their effects on primary outcome (question number 2) considered in statistical analysis?	Adjustment for age, education, socio-economic status, BMI	Low Risk of Bias High Risk of Bias Unclear

## Appendix 2

**PUBMED:**

((((((Label*[tiab] AND Food[tiab]) OR (Label*[tiab] AND Nutrition*[tiab]) OR (Menu[tiab] AND Label*[tiab]) OR (food[tiab] AND marking[tiab]) OR (food[tiab] AND packing[tiab]) OR (food[tiab] AND wrapping[tiab]) OR (Calorie*[tiab] AND Converter[tiab]) OR (Label*[tiab] And eat*[tiab]) OR (Label*[tiab] AND Nutrient*[tiab]) OR (Nutrient[tiab] AND content[tiab]) OR “food Away from home”[tiab] OR “traffic light”[tiab] OR PACE[tiab]))) AND (((Activit*[tiab] AND Motor[tiab]) OR (Activity[tiab] AND Physical[tiab]) OR (Activit*[tiab] AND Locomotor[tiab]) OR Exercise[tiab] OR (Energy[tiab] AND Expenditure[tiab]) OR walk*[tiab]))) AND (((Restriction[tiab] AND Caloric[tiab]) OR (Diet [tiab] AND “Low-Calorie”[tiab]) OR “Low Calorie Diet”[tiab] OR (Energy[tiab] AND Intake[tiab]) OR (Caloric[tiab] AND intake[tiab] AND restriction[tiab]) OR Calorie[tiab] OR kilocalorie[tiab] OR “Food energy”[tiab] OR K-Cal[tiab] OR Kcal[tiab] OR (meal*[tiab] AND “Low Calorie”[tiab]) OR (meal*[tiab] AND Low-Calorie[tiab]) OR (food[tiab] AND order[tiab]) OR (food[tiab] AND consume[tiab]) OR (food[tiab] AND consumption[tiab]) OR (food[tiab] AND decision[tiab]) OR (diet[tiab] AND selection[tiab]) OR (diet[tiab] AND decision[tiab]) OR (food[tiab] AND desire[tiab]) OR (diet[tiab] AND desire[tiab]) OR (food[tiab] AND choose[tiab]) OR (diet[tiab] AND choose[tiab]) OR (appetite[tiab] AND regulation[tiab]) OR (feed[tiab] AND intake[tiab]) OR (feeding[tiab] AND methods[tiab]) OR (food[tiab] AND ingestion[tiab]) OR (“food intake”[tiab] AND regulation[tiab]) OR (food[tiab] AND intake[tiab]) OR (food[tiab] AND uptake[tiab]) OR (Preference[tiab] AND Food[tiab]) OR (Selection*[tiab] AND Food[tiab]) OR (food[tiab] AND choice[tiab]) OR (behavior[tiab] AND eating[tiab]) OR (nutrition[tiab] AND behavior[tiab])))) AND (2000/01/01:2016/10/31[dp])

**SCOPUS:**

((((TITLE-ABS (label*) AND TITLE-ABS (food)) OR (TITLE-ABS (label*) AND TITLE-ABS (nutrition*)) OR (TITLE-ABS (menu) AND TITLE-ABS (label*)) OR (TITLE-ABS (food) AND TITLE-ABS (marking)) OR (TITLE-ABS (food) AND TITLE-ABS (packing)) OR (TITLE-ABS (food) AND TITLE-ABS (wrapping)) OR (TITLE-ABS (calorie*) AND TITLE-ABS (converter)) OR (TITLE-ABS (label*) AND TITLE-ABS (eat*)) OR (TITLE-ABS (label*) AND TITLE-ABS (nutrient*)) OR (TITLE-ABS (nutrient) AND TITLE-ABS (content)) OR TITLE-ABS (“food Away from home”) OR TITLE-ABS (“traffic light”) OR TITLE-ABS (pace))) AND (TITLE-ABS (activit*) AND TITLE-ABS (motor)) OR (TITLE-ABS (activit*) AND TITLE-ABS (physical)) OR (TITLE-ABS (activit*) AND TITLE-ABS (locomotor)) OR TITLE-ABS (exercise) OR (TITLE-ABS (energy) AND TITLE-ABS (expenditure)) OR TITLE-ABS (walk*) AND ((TITLE-ABS (restriction) AND TITLE-ABS (caloric)) OR (TITLE-ABS (diet*) AND TITLE-ABS (“Low-Calorie”)) OR TITLE-ABS (“Low Calorie Diet”) OR (TITLE-ABS (energy) AND TITLE-ABS (intake)) OR (TITLE-ABS (caloric) AND TITLE-ABS (intake) AND TITLE-ABS (restriction)) OR TITLE-ABS (calorie) OR TITLE-ABS (kilocalorie) OR TITLE-ABS (“Food energy”) OR TITLE-ABS (k-cal) OR TITLE-ABS (kcal) OR (TITLE-ABS (meal*) AND TITLE-ABS (“Low Calorie”)) OR (TITLE-ABS (meal*) AND TITLE-ABS (low-calorie)) OR (TITLE-ABS (food) AND TITLE-ABS (order)) OR (TITLE-ABS (food) AND TITLE-ABS (consume)) OR (TITLE-ABS (food) AND TITLE-ABS (consumption)) OR (TITLE-ABS (food) AND TITLE-ABS (decision)) OR (TITLE-ABS (diet) AND TITLE-ABS (selection)) OR (TITLE-ABS (diet) AND TITLE-ABS (decision)) OR (TITLE-ABS (food) AND TITLE-ABS (desire)) OR (TITLE-ABS (diet) AND TITLE-ABS (desire)) OR (TITLE-ABS (food) AND TITLE-ABS (choose)) OR (TITLE-ABS (diet) AND TITLE-ABS (choose)) OR (TITLE-ABS (appetite) AND TITLE-ABS (regulation)) OR (TITLE-ABS (feed) AND TITLE-ABS (intake)) OR (TITLE-ABS (feeding) AND TITLE-ABS (methods)) OR (TITLE-ABS (food) AND TITLE-ABS (ingestion)) OR (TITLE-ABS (“food intake”) AND TITLE-ABS (regulation)) OR (TITLE-ABS (food) AND TITLE-ABS (intake) OR (TITLE-ABS (food) AND TITLE-ABS (uptake)) OR (TITLE-ABS (preference) AND TITLE-ABS (food)) OR (TITLE-ABS (selection) AND TITLE-ABS (food)) OR (TITLE-ABS (food) AND TITLE-ABS (choice)) OR (TITLE-ABS (behavior) AND TITLE-ABS (eat*)) OR (TITLE-ABS (nutrition) AND TITLE-ABS (behavior))))) AND ((((PUBYEAR > 1999 AND PUBYEAR < 2016) OR PUBDATETXT (january 2016) OR PUBDATETXT (february 2016) OR PUBDATETXT (march 2016) OR PUBDATETXT (april 2016) OR PUBDATETXT (may 2016) OR PUBDATETXT (june 2016) OR PUBDATETXT (july 2016) OR PUBDATETXT (agust 2016) OR PUBDATETXT(September 2016) OR PUBDATETXT(October 2016))))

**Cochrane:**

((((((Label* AND Food) OR (Label* AND Nutrition*) OR (Menu AND Label*) OR (food AND packing) OR (food AND wrapping) OR (Calorie* AND Converter) OR (Label* And eat*) OR (Label* AND Nutrient*) OR (Nutrient AND content) OR “food Away from home” OR “traffic light” OR PACE))) AND (((Activit* AND Motor) OR (Activity AND Physical) OR (Activit* AND Locomotor) OR Exercise OR (Energy AND Expenditure) OR walk*))) AND (((Restriction AND Caloric) OR (Diet AND “Low-Calorie”) OR “Low Calorie Diet” OR (Energy AND Intake) OR (Caloric AND intake AND restriction) OR Calorie OR kilocalorie OR “Food energy” OR K-Cal OR Kcal OR (meal* AND “Low Calorie”) OR (meal* AND Low-Calorie) OR (food AND order) OR (food AND consume) OR (food AND consumption) OR (food AND decision) OR (diet AND selection) OR (diet AND decision) OR (food AND desire) OR (diet AND desire) OR (food AND choose) OR (diet AND choose) OR (appetite AND regulation) OR (feed AND intake) OR (feeding AND methods) OR (food AND ingestion) OR (“food intake” AND regulation) OR (food AND intake) OR (food AND uptake) OR (Preference AND Food) OR (Selection* AND Food) OR (food AND choice) OR (behavior AND eating) OR (nutrition AND behavior)))) AND (2000:2016)

**Web of science: 506**

((TI = (label*) AND TI = (food)) OR (TS = (label*) AND TS = (nutrition*)) OR (TS = (menu) AND TS = (label*)) OR (TI = (food) AND TI = (marking)) OR (TI = (food) AND TI = (packing)) OR (TI = (food) AND TI = (wrapping)) OR (TS = (calorie*) AND TS = (converter)) OR (TI = (label*) AND TI = (eat*)) OR (TI = (label*) AND TI = (nutrient*)) OR (TS = (nutrient) AND TS = (content)) OR TS = (“food Away from home”) OR TS = (“traffic light”) OR TS = (pace)) AND ((TI = (activit*) AND TI = (motor)) OR (TI = (activit*) AND TI = (physical)) OR (TI = (activit*) AND TI = (locomotor)) OR TI = (exercise) OR (TS = (energy) AND TS = (expenditure)) OR TS = (walk*)) AND ((TS = (restriction) AND TS = (caloric)) OR (TS = (diet*) AND TS = (“Low-Calorie”)) OR TS = (“Low Calorie Diet”) OR (TS = (energy) AND TS = (intake)) OR (TS = (caloric) AND TS = (intake) AND TS = (restriction)) OR TS = (calorie) OR TS = (kilocalorie) OR TS = (“Food energy”) OR TS = (k-cal) OR TS = (kcal) OR (TS = (meal*) AND TS = (“Low Calorie”)) OR (TS = (meal*) AND TS = (low-calorie)) OR (TS = (food) AND TS = (order)) OR (TS = (food) AND TS = (consume)) OR (TS = (food) AND TS = (consumption)) OR (TS = (food) AND TS = (decision)) OR (TS = (diet) AND TS = (selection)) OR (TS = (diet) AND TS = (decision)) OR (TS = (food) AND TS = (desire)) OR (TS = (diet) AND TS = (desire)) OR (TS = (food) AND TS = (choose)) OR (TS = (diet) AND TS = (choose)) OR (TS = (appetite) AND TS = (regulation)) OR (TS = (feed) AND TS = (intake)) OR (TS = (feeding) AND TS = (methods)) OR (TS = (food) AND TS = (ingestion)) OR (TS = (“food intake”) AND TS = (regulation)) OR (TS = (food) AND TS = (intake) OR (TS = (food) AND TS = (uptake)) OR (TI = (preference) AND TI = (food)) OR (TS = (selection) AND TS = (food)) OR (TS = (food) AND TS = (choice)) OR (TS = (behavior) AND TS = (eat*)) OR (TS = (nutrition) AND TS = (behavior)))) AND (PY = (2000–2016))

## Abbreviations

BMI: Body mass index
CALERIE: Comprehensive assessment of the long-term effects of reducing intake of energy
DNA: Deoxyribonucleic acid
FFM: Fat free mass
PICOS: Population, intervention, comparator, outcome, setting
RNA: Ribonucleic acid
