# Supplementary material for: Physical activity equivalent labeling vs. calorie labeling: a systematic review and meta-analysis
Source: Int J Behav Nutr Phys Act. 2018 Sep 14;15:88. doi: 10.1186/s12966-018-0720-2 (PMC6137736; doi:10.1186/s12966-018-0720-2)
Supplement: Supplementary file 1 — Table S1. PICOS (population, intervention, comparator, outcome, setting). (DOC 29 kb) [file 12966_2018_720_MOESM1_ESM.doc]

| Table S1. PICOS (population, intervention, comparator, outcome, setting) | |
| --- | --- |
| Topic | Physical activity equivalent labeling vs. calorie labeling: A systematic review and meta-analysis |
| Population | Adults ( ≤18 years) |
| Intervention | Physical activity equivalent labeling and calorie labeling |
| Comparison | The effects of Physical activity equivalent labeling vs. calorie labeling on calorie order |
| Outcome | Calorie order |
| Setting | Trials and Observational studies with any design which was either population based or non-population based in restaurant, schools or other settings are included. Qualitative studies are also assessed. |
